# Dendritic distribution of autophagosomes underlies pathway-selective induction of LTD

**DOI:** 10.1016/j.celrep.2023.112898

**Published:** 2023-07-28

**Authors:** Kevin M. Keary, Qin-Hua Gu, Jiji Chen, Zheng Li

**Affiliations:** 1 Section on Synapse Development Plasticity, National Institute of Mental Health, National Institutes of Health, Bethesda, MD 20892, USA; 2 Department of Neuroscience, Brown University, Providence, RI 02912, USA; 3 Advanced Imaging and Microscopy (AIM) Resource, National Institute of Biomedical Imaging and Bioengineering, National Institutes of Health, Bethesda, MD 20892, USA; 4 Lead contact

## Abstract

The mechanism of long-term depression (LTD), a cellular substrate for learning, memory, and behavioral flexibility, is extensively studied in Schaffer collateral (SC) synapses, with inhibition of autophagy identified as a key factor. SC inputs terminate at basal and proximal apical dendrites, whereas distal apical dendrites receive inputs from the temporoammonic pathway (TAP). Here, we demonstrate that TAP and SC synapses have a shared LTD mechanism reliant on NMDA receptors, caspase-3, and autophagy inhibition. Despite this shared LTD mechanism, proximal apical dendrites contain more autophagosomes than distal apical dendrites. Additionally, unlike SC LTD, which diminishes with age, TAP LTD persists into adulthood. Our previous study shows that the high autophagy in adulthood disallows SC LTD induction. The reduction of autophagosomes from proximal to distal dendrites, combined with distinct LTD inducibility at SC and TAP synapses, suggests a model where the differential distribution of autophagosomes in dendrites gates LTD inducibility at specific circuits.

## In brief

Keary III et al. find that distinct circuits synapsing onto different parts of the same CA1 apical dendrite require autophagy inhibition for LTD. There are differing profiles of LTD inducibility correlating with a gradient of autophagosomes from proximal to distal dendrites. Autophagosome density may be a factor determining LTD inducibility.

## Graphical Abstract

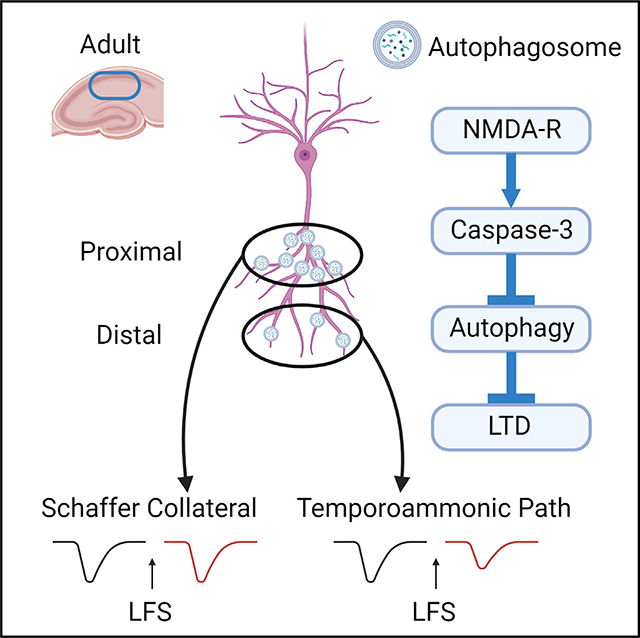

## INTRODUCTION

Alteration of synaptic strength via synaptic plasticity is a cellular model for learning, memory, and adaptive behavior.^1^ Dysfunctional synaptic plasticity is associated with many psychiatric and neurological disorders.^2^ Synaptic plasticity can last for hours and up to days and months. This long-term synaptic plasticity can be further subdivided into long-term potentiation (LTP) and long-term depression (LTD), the strengthening and weakening of synapses, respectively.^3^ The mechanism by which the strength of excitatory synapses changes is largely through alteration of surface α-amino-3-hydroxy-5-methyl-4-isoxazolepropionic acid (AMPA) receptors, either inserted into the cell membrane in the case of LTP or removed from the membrane in LTD.^4–6^ LTD, while comparatively less studied than LTP, is an equally important component of long-term synaptic plasticity. LTD has been shown to be involved in hippocampus-dependent functions such as spatial navigation, behavioral flexibility, and fear memory.^7–9^

The hippocampal formation is a group of structures consisting of the dentate gyrus (DG), *cornu ammonis* (CA1–CA3), subiculum, and entorhinal cortex (EC).^10^ The connectivity of the hippocampus is extensively defined, with the major input to the hippocampus being layer II neurons of the EC projecting to the DG. The DG sends mossy fibers to CA3, and CA3 projects Schaffer collaterals (SCs) to the *stratum radiatum* (SR) and *stratum oriens* (SO) of CA1, which serves as the primary output structure of the hippocampus.^10–12^ Layer III neurons of the EC also send a direct projection to the distal apical dendrites in the *stratum lacunosum moleculare* (SLM) of CA1 via the temporoammonic pathway (TAP).^13^ The TAP carries projections from the reuniens nucleus of the thalamus (NeR) in addition to the direct cortical input from the EC.^14^ Hence, different dendritic segments in the same CA1 neuron receive synaptic inputs from different neural circuits. Synaptic transmission from different neural circuits is not necessarily modulated concurrently even in the same dendrite. Circuit-selective synaptic plasticity can enhance the capacity and flexibility of the nervous system. Dendritic segment-specific synaptic properties in CA1 neurons, such as differences in synapse number, AMPAR abundance, and synaptic structure, have already been noted.^15,16^ However, the mechanism for circuit-selective control of synaptic plasticity in the same dendrite is largely unclear.

Induction of LTD by low-frequency stimulation (LFS) at the SC synapse requires caspase-3 and inhibition of autophagy.^17–19^ LFS leads to N-methyl-D-aspartate receptor (NMDAR) activation and calcium influx, which activates protein phosphatases, including calcineurin and PP1.^20,21^ The phosphatases dephosphorylate the pro-apoptotic BCL-2-family protein BAD to activate caspase-3, which cleaves autophagy-related proteins, resulting in autophagy inhibition.^18,22^ Autophagy inhibition leads to a reduction in AMPA receptor recycling to the plasma membrane, thereby decreasing surface AMPA receptor levels.^17^ This NMDAR-dependent LTD (NMDAR-LTD) is readily inducible in juvenile mice (post-natal day 16 [P16]–P19) but is difficult to induce in adult mice (>P56).^23^ This developmental shift in LTD inducibility is in part attributable to autophagy upregulation, as autophagy in CA1 is increased in adults compared with juvenile mice, and knocking out autophagy-related proteins restores SC LTD inducibility in adult mice.^17^

LTD at the TAP synapse remains poorly understood, with inconsistent data in the literature. Some studies indicate that LTD in the TAP can be induced in adult rats in an NMDAR-dependent fashion.^24^ Other work is contradictory, showing that TAP LTD is NMDAR independent.^25^ Little is known about the intracellular signaling cascade for TAP LTD. This stands in stark contrast to the extensively characterized SC LTD. In this study, we investigate the mechanism for TAP LTD and propose an autophagosome-based model for the differential LTD inducibility of distinct dendritic segments.

## RESULTS

### Autophagosomes decrease in density with distance along CA1 apical dendrites

**Figure 1.**
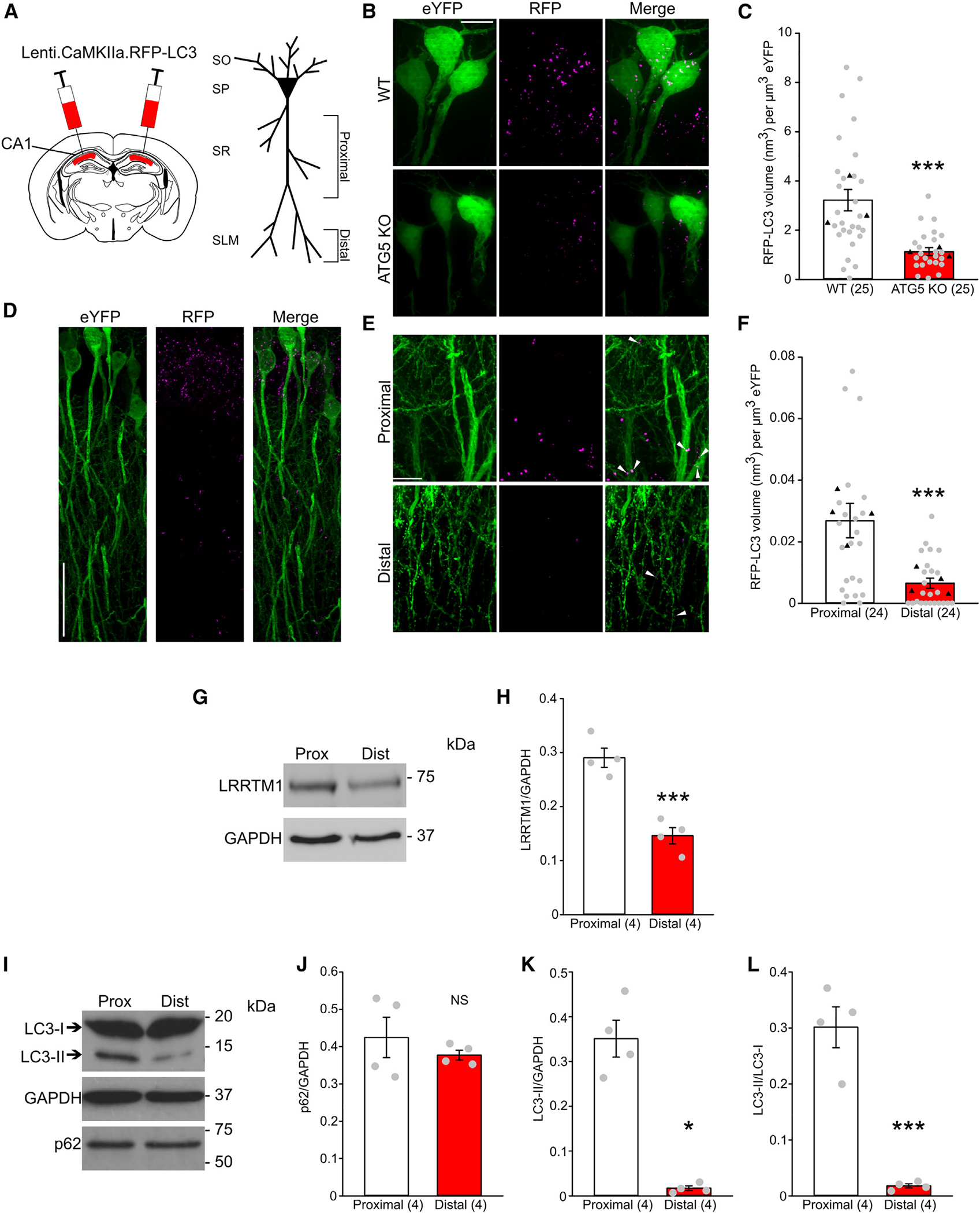
The autophagosome abundance is higher in proximal apical dendrites than in distal apical dendrites in CA1 neurons (A) Diagram of virus injection (left) and diagram of a CA1 pyramidal neuron (right) with CA1 subregions and proximal and distal apical dendrites labeled. (B) Representative iSIM images of CA1 pyramidal neurons from THY1-eYFP-ATG5^flox/flox^ (WT) and THY1-eYFP-ATG5^flox/flox^ Cre (ATG5 KO) mice injected with the RFP-LC3 virus; scale bar, 10 μm. (C) Quantification of RFP-LC3 puncta volume in eYFP-labeled neurons; n = 25 images from 3 animals, with black triangles denoting animal means. (D) Representative confocal images of apical CA1 dendritic arbors of THY1-eYFP mice injected with the RFP-LC3 virus; scale bar, 50 μm. (E) Representative iSIM images taken from the CA1 proximal and distal apical dendrites of THY1-eYFP mice injected with the RFP-LC3 virus. White arrowheads indicate colocalized RFP-LC3 puncta in eYFP-labeled dendrites; scale bar, 10 μm. (F) Quantification of RFP-LC3 puncta volume in eYFP-labeled proximal and distal dendrites; n = 24 images from 4 animals, with black triangles denoting animal means. (G) Representative blots of tissue lysates taken from the CA1 proximal and distal dendrites for assessing LRRTM1. (H) Quantification of LRRTM1 in proximal and distal lysates; n = 4 biological replicates. (I) Representative blots of tissue lysates taken from the CA1 proximal and distal dendrites for assessing autophagic flux. (J–L) Quantification of autophagic flux in western blots for proximal and distal lysates; n = 4 biological replicates. Data are presented as mean ± SEM. *p < 0.05, ***p < 0.001.

Our previous studies have shown that autophagy inhibition by caspase-3 is required for induction of NMDAR-LTD in the SC pathway, which terminates at the basal and proximal apical dendrites of CA1.^11,17^ In addition to the SC pathway, the TAP projects to CA1, terminating at the distal apical dendrites within the SLM. To test whether autophagy is involved in LTD of the TAP, we first examined whether autophagosomes are present in the distal apical dendrites of CA1 neurons. To this end, we generated a lentivirus expressing RFP-LC3 under control of the Ca^2+^/calmodulin-dependent protein kinase II (CaMKII) promoter and injected virus into the CA1 region of P28 Thy1-eYFP-H mice, a line expressing eYFP in a subset of hippocampal pyramidal neurons (Figure 1A and S1A).^26,27^ LC3 is a widely used marker of autophagosomes in imaging studies.^28,29^ Four weeks post injection, brain sections were prepared from injected mice for imaging. Because autophagosomes range from 0.3 to a few micrometers in diameter, which is close to the diffraction limit of conventional confocal microscopy, we used structured illumination microscopy (iSIM) for acquisition of high-magnification images.^30,31^ To ensure that only autophagosomes in CA1 pyramidal neurons were assessed, a custom Fiji script was used to measure RFP puncta colocalized with eYFP.

To validate RFP-LC3 as an autophagosome marker, we crossed Thy1-eYFP-H mice with CA1 and forebrain excitatory neuron-specific ATG5 knockout mice (ATG5^flox/flox^-T29-Cre). Immunostaining for ATG5 revealed significant ATG5 decreases in the CA1 and EC of the knockout mice (Figures S1B–S1E). Autophagic flux in the CA1 and EC lysates from the knockout mice was assessed by western blotting for LC3-II and p62, an autophagy substrate. Autophagic flux was reduced as indicated by a p62 increase and LC3-II decrease in EC and CA1 lysates from ATG5 knockout (KO) animals compared with wild-type controls (Figures S1F–S1M). Hence, autophagy is reduced in the CA1 and EC of this mouse line. RFP-LC3 puncta volume in eYFP-positive neurons was markedly reduced in CA1 cell bodies of Thy1-eYFP-ATG5^flox/flox^-T29-Cre mice compared with wild-type littermates, validating RFP-LC3 as an autophagosome marker (RFP-LC3 volume per 1 μm^3^ eYFP: 1.128 ± 0.157 nm^3^ in KO mice and 3.218 ± 0.435 nm^3^ in wild-type littermates, p < 0.001; Figures 1B and 1C). We then imaged proximal apical dendrites (25–150 μm from the soma) and distal apical dendrites (>200 μm from the soma) in Thy1-eYFP-H mice injected with the RFP-LC3 virus (Figures 1D and 1E). eYFP-positive proximal apical dendrites had a greater colocalized RFP-LC3 puncta volume than eYFP-positive distal dendrites (RFP-LC3 volume per 1 μm^3^ eYFP: 2.69 × 10^−2^ ± 5.58 × 10^−3^ nm^3^ in proximal dendrites and 6.563 × 10^−3^ ± 1.665 × 10^−3^ nm^3^ in distal dendrites, p < 0.001; Figure 1F).

To further assess autophagy in proximal and distal dendrites, we dissected out the CA1 regions containing proximal apical dendrites and distal apical dendrites. To validate separation of proximal and distal dendrites, western blotting was conducted for LRRTM1, a synaptic adhesion molecule abundant in CA1 proximal dendrites but greatly reduced in distal dendrites.^32^ Our proximal lysates had ~2-fold more LRRTM1 than distal lysates (Figures 1G and 1H), indicating that these lysates contain selective dendritic segments. The proximal apical dendrite lysate had more LC3-II than the distal apical dendrite lysate (Figures 1I, 1K, and 1L). Interestingly, no difference in p62 was detected between proximal and distal lysates (Figures 1I and 1J). This could be attributed to the fact that our lysates lack *stratum pyramidale* (the soma-containing layer where autophagosome cargo is degraded^33^) and contain non-pyramidal cells, which may have similar levels of autophagic degradation in the SR and SLM. These findings indicate that there are fewer autophagosomes in distal apical dendrites than in proximal apical dendrites.

### TAP LTD persists in adulthood in an NMDAR-dependent fashion

**Figure 2.**
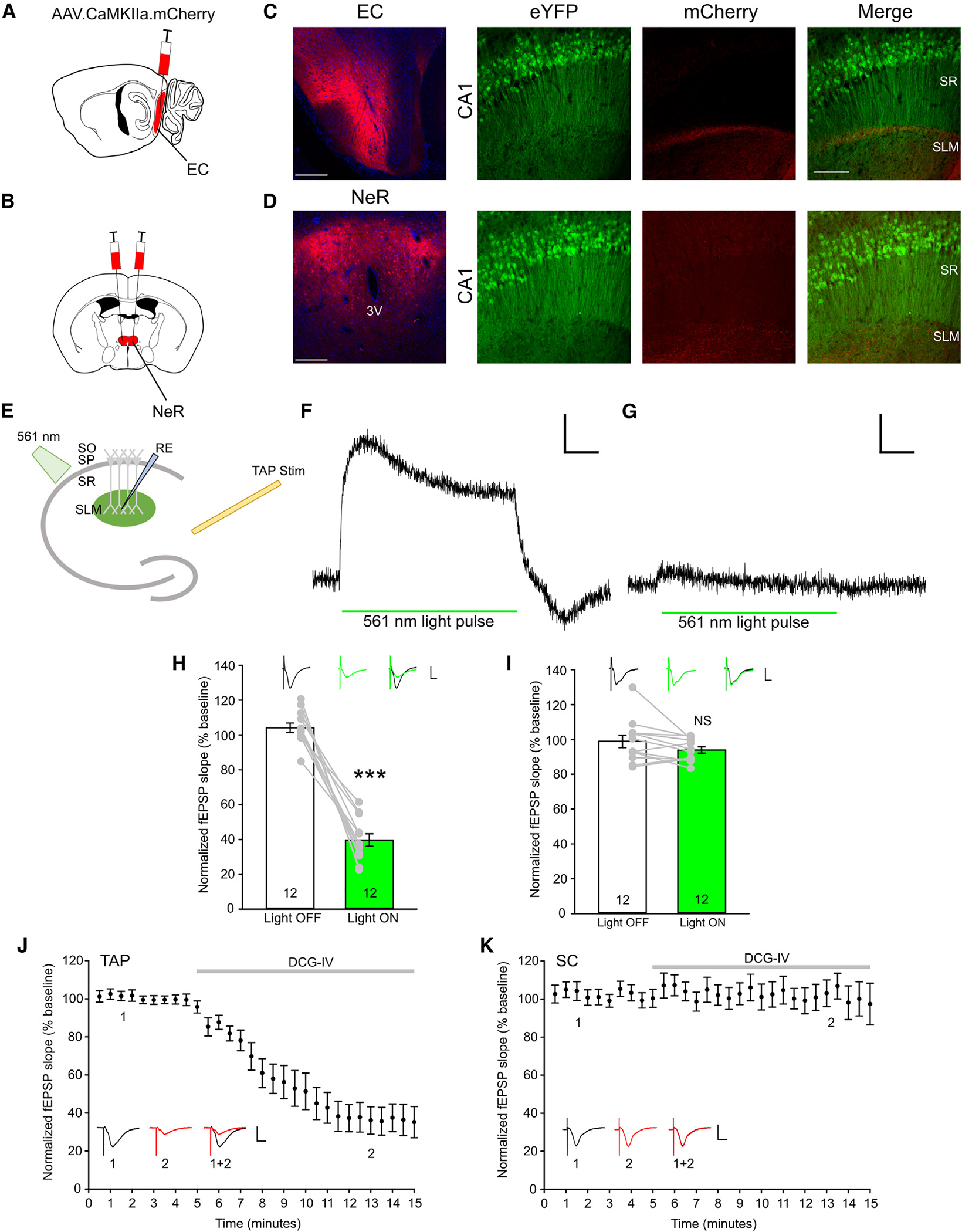
Validation of TAP electrical stimulation in modified horizontal slices (A and B) Diagrams of virus injection into the EC (A) and NeR (B). (C) Representative confocal images of the injection site at the EC (left) and projections terminating at CA1 (right) in modified horizontal slices from THY1-eYFP animals. (D) Representative confocal images of the injection site at the NeR, with the third ventricle labeled as 3V (left), and projections terminating at CA1 (right) in modified horizontal slices from THY1-eYFP animals. Scale bars, 200 μm (left) and 100 μm (right). (E) Schematic of co-application of optical and electrical stimulation in slices from mice injected with the ArchT-GFP virus. (F) (F and G) Representative traces of optically evoked potentials by 100-ms 561-nm light application in EC injected (F) and NeR injected (G) slices. Scale bars, 0.1 mV (vertical) and 20 ms (horizontal). (G) (H and I) Quantification of electrically evoked normalized field excitatory postsynaptic potentials (fEPSPs) under Light OFF and Light ON conditions in EC injected and NeR injected (I) slices; each data point represents the averaged responses evoked by stimulation applied once every 30 s during a period of 10 min. Black trace, Light OFF; green trace, Light ON; scale bars, 0.5 mV (vertical) and 20 ms (horizontal). n = 12 slices from 7 animals (H) and 12 slices from 6 animals (I). (H) (J and K) Normalized fEPSP slopes evoked by stimulating the TAP (J) and SC (K) before and after perfusion with 100 μM DCG-IV; scale bars, 0.5 mV (vertical) and 20 ms (horizontal); n = 10 slices from 3 animals. Each data point represents average responses from all slices of 6 consecutive traces evoked every 5 s. Data are presented as mean ± SEM. ***p < 0.001.

The TAP consists of inputs from layer III of the EC and NeR, terminating at the distal apical dendrites of CA1.^12,34,35^ To examine LTD in the TAP, we used an angled horizontal slice preparation to preserve this pathway for electrophysiology.^36–38^ To determine whether our slicing protocol indeed preserves the TAP, we injected an adeno-associated virus (AAV) expressing mCherry controlled by the CaMKII promoter into the EC and NeR, the two regions projecting via the TAP, of Thy1-eYFP mice (Figures 2A and 2B). mCherry-expressing cells in the injected region were positive for CaMKII, indicating that our virus specifically transduces excitatory neurons (Figure S2A). The percentage of cells transduced by virus at the EC and NeR was comparable between the two areas (Figures S2B and S2C). mCherry-labeled axons were readily detected in the CA1 SLM of horizontal slices in EC-injected mice, with very few seen in NeR-injected mice (Figures 2C and 2D). The presence of EC and NeR axons in the slices indicates that our slicing protocol preserved the TAP.

We next sought to confirm that our electrical stimulation is stimulating TAP axons. If so, optically silencing projections from the EC and NeR should reduce the electrically evoked field excitatory postsynaptic potentials (fEPSPs). To test this, we injected an AAV expressing ArchaerhodopsinT-GFP (ArchT) under the CaMKII promoter into the NeR or EC of P28 wild-type mice and then prepared angled horizontal slices 6 weeks post injection. ArchT is a light-gated outward proton pump capable of presynaptic silencing by increasing the presynaptic pH to inhibit vesicular release.^39^ 561-nm optical stimulation was applied to the slice to activate ArchT in EC and NeR axons directly projecting to CA1 via the TAP (Figure 2E). Positive optical potentials resulting from the flow of hydrogen ions into the extracellular space through ArchT in response to light were detected under EC and NeR conditions. While slices from both injection conditions responded to optical stimulation, there were greater optically evoked potentials in the EC-injected slices than in the NeR-injected slices (Figures 2F and 2G).

To determine whether electrical stimulation recruits TAP axons, we co-delivered electrical stimulation to the TAP and 561-nm light pulses in slices injected with the ArchT-GFP virus in the EC or NeR. In the EC injected slices, the normalized fEPSP slope was significantly reduced in the presence of light (Light OFF, 104.103 ± 2.75; Light ON, 39.581 ± 3.578; p = 1.29 × 10^−7^; Figure 2H). In the NeR injected slices, there was a trending decrease in normalized fEPSP slope, but it was not statistically significant (Light OFF, 98.917 ± 3.603; Light ON, 93.902 ± 1.824; p = 0.064; Figure 2I). Additionally, normalizing the ArchT-induced fEPSP change to the optical potential suggests that EC ArchT has a greater degree of inhibition than NeR ArchT (Figure S2D). To further validate TAP stimulation in our recording paradigm, we applied the group II metabotropic glutamate receptor (mGluR) agonist (2*S*,2′*R*,3′*R*)-2-(2′,3′-dicarboxycyclopropyl) glycine (DCG-IV), a drug shown previously to block TAP but not SC inputs, to our slices.^40^ DCG-IV application significantly reduced fEPSPs evoked by stimulating the TAP but not by stimulating SC (Figures 2J and 2K). The optogenetics and pharmacological experiments indicate that our electrical stimulation in the angled horizontal slices specifically recruits TAP axons.

**Figure 3.**
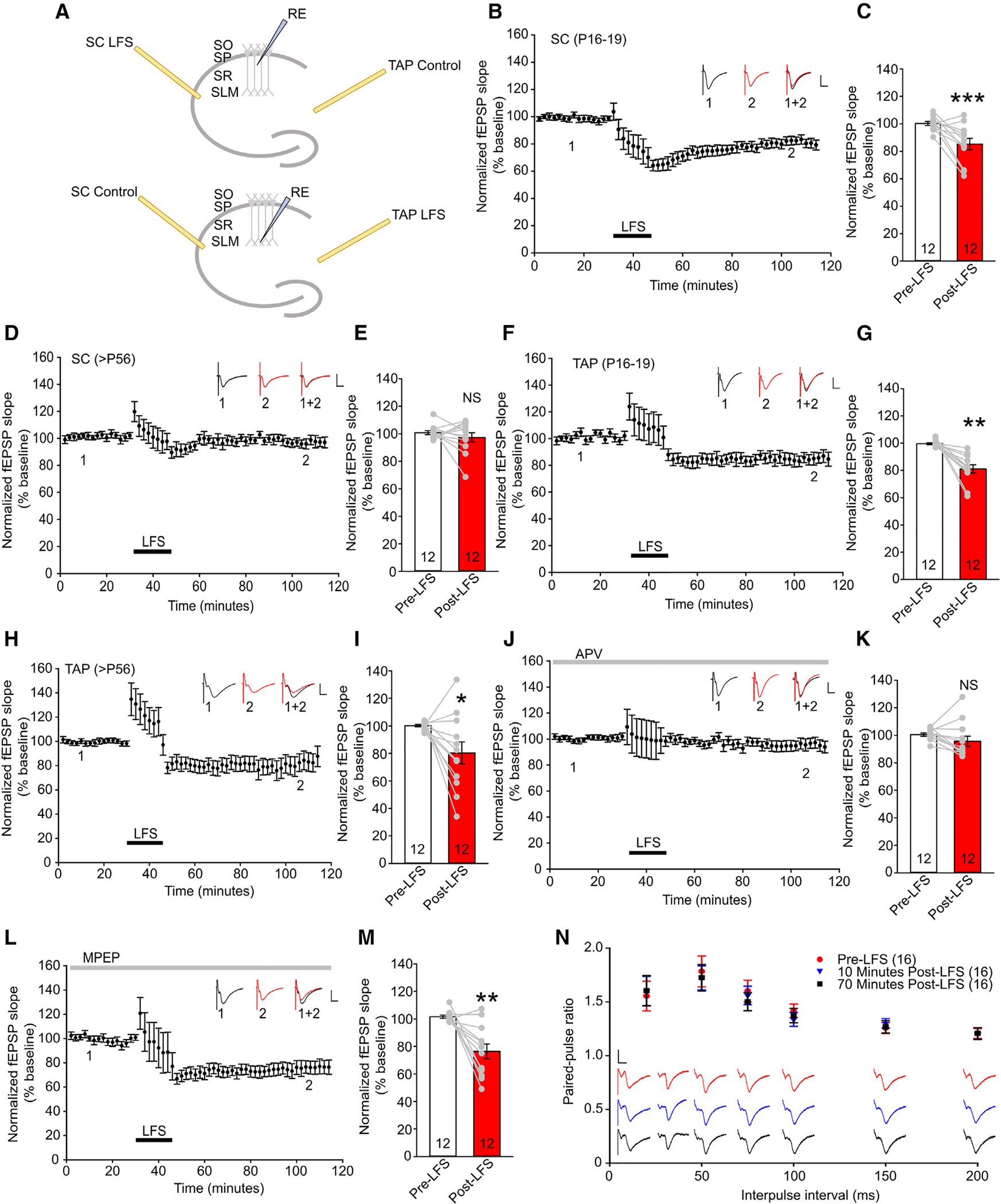
TAP LTD persists into adulthood in an NMDAR-dependent fashion (A) Schematic of stimulating and recording electrode (RE) placement in the SC (top) and TAP (bottom) paradigms. (B) Normalized fEPSPs evoked by stimulating SC in young slices. (C) Quantification of normalized fEPSP slopes in young SC slices before and after LFS; n = 12 slices from 8 animals. (D and E) Normalized fEPSPs evoked by stimulating SC in adult slices and quantification of fEPSPs before and after LFS; n = 12 slices from 10 animals. (F and G) Normalized fEPSPs evoked by stimulating the TAP in young slices and quantification of fEPSPs before and after LFS; n = 12 slices from 7 animals. (H and I) Normalized fEPSPs evoked by stimulating the TAP in adult slices and quantification of fEPSPs before and after LFS; n = 12 slices from 12 animals. (J and K) Normalized fEPSPs evoked by stimulating the TAP in adult slices in the presence of 50 μM APV and quantification of fEPSPs before and after LFS; n = 12 slices from 9 animals. (L and M) Normalized fEPSPs evoked by stimulating the TAP in adult slices in the presence of 10 μM MPEP and quantification of fEPSPs before and after LFS; n = 12 slices from 10 animals. Each data point in (B), (D), (F), (H), (J), and (L) represents averaged response from all slices of 4 consecutive traces evoked every 30 s; scale bars, 0.5 mV (vertical) and 20 ms (horizontal). Each data point in (C), (E), (G), (I), (K), and (M) represents the average of the first 10 min of pre-LFS and last 10 min of post-LFS from each slice. (N) Paired-pulse ratio (PPR), measured by stimulating the TAP pre-LFS, 10 min post-LFS, and 70 min post-LFS. Each data point represents the averaged PPR from 16 slices of 6 animals. Data are presented as mean ± SEM. *p < 0.05, **p < 0.01.

Having confirmed the TAP electrical stimulation paradigm in our preparations, we applied LFS (900 electrical pulses at 1 Hz) to induce LTD. For SC, fEPSPs were recorded from the SR with LFS applied to the SC electrode and the TAP electrode serving as the uninduced control. For TAP LTD, fEPSPs were recorded from the CA1 SLM with LFS applied to the TAP electrode and the SC electrode serving as the uninduced control (Figure 3A). Slices with more than a 10% change in fEPSP slopes in the uninduced pathway from pre-induction baseline during the recording were excluded. While the stimulating electrodes were about 1 mm apart and unlikely to stimulate the same input, to ensure that the electrodes were selectively recruiting the TAP and SC pathways independently, we conducted an independent-input test. A single pulse was delivered to each electrode sequentially with an interpulse interval of 50 ms. This was followed by another pair of pulses delivered to the two electrodes with the inverse sequence. The ratio of pulses 1 and 2 from the same input should not exhibit any short-term plasticity if the two electrodes stimulate independent inputs. Any slices failing the independent-input test were excluded from analysis.

LTD was readily inducible in young (P16–P19) SC slices (normalized fEPSP slopes: 99.55 ± 0.68 for pre-LFS, 81.17 ± 3.03 for post-LFS, p < 0.001; Figures 2B and 2C) but abolished in adult (>P56) SC slices (normalized fEPSP slopes: 100.84 ± 1.41 for pre-LFS, 97.34 ± 3.39 for post-LFS, p = 0.386; Figures 3D and 3E). Conversely, LFS of the TAP readily induced LTD in young and adult slices (normalized fEPSP slopes: 100.27 ± 1.45 for pre-LFS, 85.29 ± 4.08 for post-LFS, p = 0.00349 [Figures 3F and 3G]; 100.16 ± 0.90 for pre-LFS, 80.36 ± 7.95 for post-LFS, p = 0.0335 [Figures 3H and 3I]). TAP LTD was abolished by application of the NMDAR antagonist D-(−)-2-amino-5-phosphonopentanoic acid (APV) (normalized fEPSP slopes: 100.48 ± 1.16 for pre-LFS, 95.64 ± 3.73 for post-LFS, p = 0.166; Figures 3J and 3K) but not the mGluR antagonist 2-methyl-6-(phenylethynyl)-pyridine hydrochloride (MPEP) in adult slices (normalized fEPSP slopes: 101.58 ± 1.18 for pre-LFS, 76.39 ± 5.39 for post-LFS, p = 1.53 × 10^−3^; Figure 3L–3M). The paired-pulse ratio (PPR) was unchanged after TAP LTD induction, indicating that TAP LTD is mediated by post-synaptic alterations (Figure 3N). These findings indicate that LFS of the TAP induces NMDAR-LTD via post-synaptic alterations.

### TAP LTD is reliant on caspase-3 and autophagy inhibition

**Figure 4.**
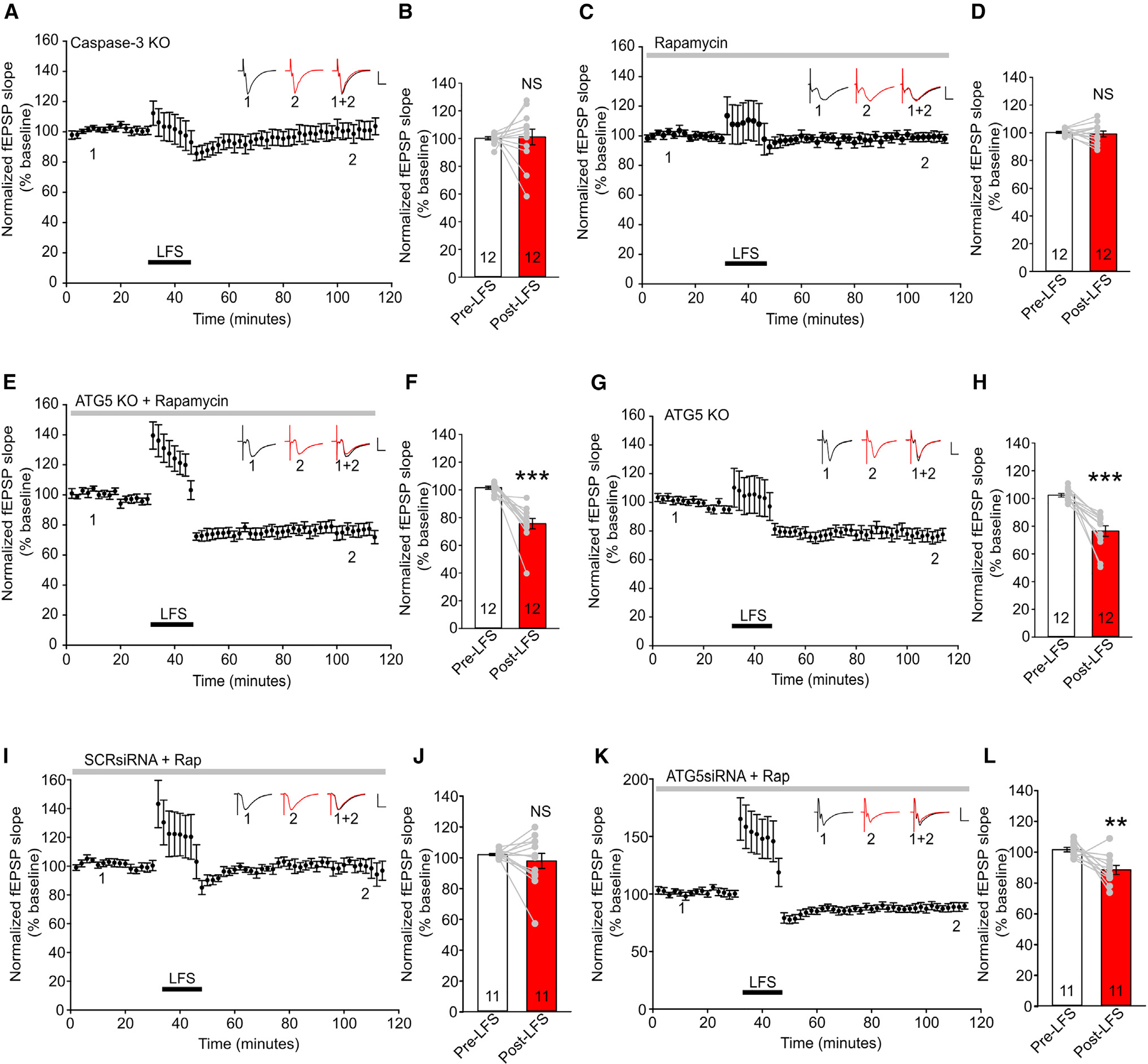
TAP LTD is reliant on caspase-3 and autophagy inhibition (A) Normalized fEPSPs evoked in adult caspase-3 KO slices. (B) Quantification of normalized fEPSP slopes in caspase-3 KO slices before and after LFS; n = 12 slices from 7 animals. (C and D) fEPSPs evoked in adult wild-type slices in the presence of 1 μM rapamycin and quantification of fEPSPs before and after LFS; n = 12 slices from 8 animals. (E and F) fEPSPs evoked in adult ATG5 KO slices in the presence of 1 μM rapamycin and quantification of fEPSPs before and after LFS; n = 12 slices from 8 animals. (G and H) fEPSPs evoked in adult ATG5 KO slices and quantification of fEPSPs before and after LFS; n = 12 slices from 9 animals. (I and J) fEPSPs evoked in adult SCRsiRNA-injected slices in the presence of 1 μM rapamycin and quantification of fEPSPs before and after LFS; n = 11 slices from 5 animals. (K and L) fEPSPs evoked in adult ATG5siRNA-injected slices in the presence of 1 μM rapamycin and quantification of fEPSPs before and after LFS; n = 11 slices from 4 animals. Each data point in (A), (C), (E), (G), (I), and (K) represents the averaged response from all slices of 4 consecutive traces evoked every 30 s; scale bars, 0.5 mV (vertical) and 20 ms (horizontal). Each data point in (B), (D), (F), (H), (J), and (L) represents the average of the first 10 min of pre-LFS and last 10 min of post-LFS from each slice. Data are presented as mean ± SEM. **p < 0.01 ***p < 0.001.

Because the TAP synapse is capable of NMDAR-dependent LTD in adult slices, we next tested whether the previously identified intracellular cascade in SC LTD of caspase-3 activation and autophagy inhibition is conserved in TAP LTD. To determine whether caspase-3 is involved in TAP LTD, we utilized caspase-3 KO mice shown previously to have impaired SC LTD.^19^ TAP LTD was present in wild-type littermates of caspase-3 KO mice (normalized fEPSP slopes: 101.58 ± 0.98 for pre-LFS, 79.51 ± 4.59 for post-LFS, p = 0.000701; Figures S3A and S3B) but abolished in caspase-3 KO mice (normalized fEPSP slopes: 100.14 ± 1.13 for pre-LFS, 101.14 ± 5.72 for post-LFS, p = 0.879; Figures 4A and 4B). The PPR and input-output relationship were unchanged in caspase-3 KO mice (Figures S3C and S3D).

Because caspase-3 inhibits autophagy in SC LTD,^17^ we proceeded to test whether the same mechanism acts on TAP LTD. Our previous work has shown that autophagy increases in the hippocampal lysate of caspase-3 KO mice.^41^ Consistent with this, iSIM imaging showed that the total volume of LC3 puncta increased in the CA1 distal apical dendrites of caspase-3 KO mice (Figures S3E and S3F), suggesting that caspase-3 inhibits autophagy in distal dendrites. To ascertain whether autophagy inhibition is involved in TAP LTD, we perfused wild-type slices with rapamycin, which acts on the mTOR pathway to increase autophagy.^42,43^ TAP LTD was abolished by bath application of rapamycin (normalized fEPSP slopes: 100.31 ± 0.71 for pre-LFS, 99.16 ± 2.13 for post-LFS, p = 0.589; Figures 4C and 4D).

Rapamycin has non-autophagy functions as well, such as promoting protein synthesis, a process known to be important for long-term synaptic plasticity.^44–47^ To test whether the effect of rapamycin is mediated by acting on autophagy, we prepared slices from the ATG5^flox/flox^-T29-Cre mice. The LTD-blocking effect of rapamycin was absent in ATG5 KO slices (normalized fEPSP slopes: 101.59 ± 1.12 for pre-LFS, 75.62 ± 3.77 for post-LFS, p = 8.83 × 10^−5^; Figures 4E and 4F). ATG5 KO slices perfused with standard artificial cerebrospinal fluid (ACSF) exhibited significant LTD (normalized fEPSP slopes: 102.33 ± 2.88 for pre-LFS, 76.49 ± 3.82 for post-LFS, p < 0.001; Figures 4G and 4H), as did wild-type littermates of ATG5 KO mice (normalized fEPSP slopes: 98.60 ± 0.75 for pre-LFS, 77.02 ± 6.55 for post-LFS, p = 0.00784; Figures S3G and S3H). TAP LTP was readily induced in ATG5 KO wild-type littermate slices (normalized fEPSP slopes: 100.48 ± 0.88 for pre-HFS, 122.84 ± 3.83 for post-HFS, p = 0.00023; Figures S3I and S3J) and ATG5 KO slices (normalized fEPSP slopes: 95.49 ± 2.67 for pre-HFS, 138.10 ± 77.33 for post-HFS, p = 0.000235; Figures S3K and S3L). This is aligned with our previous work indicating that the SC pathway in ATG5 KO mice has intact LTP.^17^ The PPR and input-output relationship were also unchanged in conditional ATG5 KO slices (Figures S3C and S3D).

Because ATG5 is knocked out in presynaptic EC neurons and postsynaptic CA1 neurons in conditional KO mice, to specifically probe post-synaptic reliance of autophagy inhibition in TAP LTD, we knocked down ATG5 in CA1 neurons by injecting a lentivirus expressing ATG5 small interfering RNAs (siRNAs) and GFP (Figure S4A). We have validated the efficiency and specificity of ATG5 siRNAs in our previous work.^17^ Scrambled (SCR) siRNA-and ATG5siRNA-injected slices exhibited LTD (normalized fEPSP slopes: 100.97 ± 1.90 for pre-LFS, 83.47 ± 5.46 for post-LFS, p = 0.0211 [Figures S4B and S4C]; normalized fEPSP slopes: 98.18 ± 0.84 for pre-LFS, 86.63 ± 3.00 for post-LFS, p = 0.00355 [Figures S4D and S4E]). Rapamycin application abolished LTD induction in SCRsiRNA-injected slices (normalized fEPSP slopes: 102.16 ± 0.67 for pre-LFS, 98.01 ± 4.92 for post-LFS, p = 0.397; Figures 4I and 4J), while LTD persisted in ATG5siRNA slices perfused with rapamycin (normalized fEPSP slopes: 101.60 ± 1.51 for pre-LFS, 88.44 ± 2.93 for post-LFS, p = 0.00844; Figures 4K and 4L). These results indicate that rapamycin acts on autophagy in postsynaptic neurons to inhibit LTD.

Taken together, these findings indicate that TAP LTD has the same induction mechanism as SC LTD, reliant on NMDA receptors, caspase-3 activation, and autophagy inhibition. The correlation between the local abundance of dendritic autophagosomes and LTD inducibility raises the possibility that the differential distribution of autophagosomes in CA1 apical dendrites underlies pathway-dependent induction of LTD.

## DISCUSSION

While synaptic plasticity has been extensively studied in the hippocampus, little is known about how the strength of synaptic transmission from different neural circuits terminating on the same dendrite can be modulated independently. We investigated this question by examining LTD of the SC and TAP inputs, which terminate at different dendritic segments of CA1 pyramidal neurons. While SC LTD has been extensively studied, the mechanism of TAP LTD is largely unclear. We demonstrate that LFS can induce TAP LTD. Additionally, the mechanism of LTD in the two synapses is shared because both are reliant on NMDAR, caspase-3, and the inhibition of autophagy.

While examining autophagosomes in dendrites, we noted that there is a decrease in autophagosomes from proximal to distal CA1 apical dendrites. Proximal apical dendrites have a density of autophagosomes four times greater than distal apical dendrites. The difference in autophagosome density can be attributed to numerous factors. One potential factor is diminishment of the endoplasmic reticulum, which donates membranes for autophagosome production, from the soma to distal dendrites.^48,49^ Given that autophagy has to be inhibited to induce LTD, the relatively low abundance of autophagosomes in distal dendrites could be more favorable for LTD induction than the comparatively high abundance of autophagosomes in proximal dendrites.

Interestingly, while LC3-II abundance decreases from proximal to distal dendrites, the p62 level does not. One possible explanation for this is that degradation of dendritic autophagosomes appears to take place predominantly in the soma. Indeed, blocking autophagosome-lysosome fusion only increases somatic autophagosomes.^50^ This is related to the subcellular distribution pattern of lysosomes, organelles required for autophagic degradation. Within dendrites, more lysosomes are present in dendrites closer to the soma than dendrites farther away. There is also a reduction in lysosomal degradation activity with increasing distance from the soma. Lysosomes toward the periphery of the soma have a reduced acidification ability, and lysosomes in more distal dendrites have reduced cathepsins, the lysosomal proteases.^33,51,52^ The tissue we isolated for p62 analysis does not include the pyramidal soma layer. This means little, if any, autophagosome degradation is taking place in these tissues, and therefore the proximal and distal samples have no difference in p62 levels. An additional contributor to p62 in tissue lysates could be autophagy occurring in non-pyramidal cells that are a part of the lysate. Because the lysate of CA1 encompasses all cell types, the impact of glia and interneurons cannot be fully discounted when interpreting western blot results.

Our model of how autophagy acts on LTD relies on inhibition of autophagy.^17^ Previous work from Kallergi et al.^53^ offers a different perspective. They show that autophagosomes increase in number and activity during LTD, which, in turn, contributes to the reduction of surface AMPAR and PSD-95. It must be noted that there are some vital differences between this study and the work from our lab. We used a standard LFS protocol (900 pulses at 1 Hz) and ACSF for LTD induction, while Kallergi et al.^53^ utilized a stronger LFS protocol (1,200 pulses at 1.4 Hz) in tandem with ACSF containing half the magnesium concentration as ours. Previous work from the same group shows that this protocol also induces changes to the PPR,^54^ something that we and others using 900 pulses at 1 Hz do not see.^55^ It is possible that the stronger LFS protocol and lower magnesium concentration used by Kallergi et al.^53^ induces LTD via different intracellular signaling or through a different time course of the same signaling. It is also noted that the autophagy decrease we detected previously following LFS is transient and at early stages of LTD. The stronger LFS protocol used by Kallergi et al.^53^ may abbreviate the transient decrease phase, making it not readily detectable. They relied on immunostaining instead of live imaging to measure LC3-labeled autophagosomes, so the initial transient decrease may have escaped detection. It is possible that autophagy contributes to different phases of LTD. An early transient decrease in autophagy, functioning to reduce AMPAR recycling, as demonstrated previously in our studies, does not exclude the later increase in autophagy to assist with degradation of AMPAR and PSD-95 as shown by Kallergi et al.^17,53^ Additionally, we found previously that autophagy inhibition is induced by LFS but not the high-frequency stimulation (HFS) that induces LTP. Kallergi et al.^53^ did not investigate autophagosomes in response to HFS. This leaves some ambiguity as to whether the autophagy increase is specifically induced by LFS or results from synaptic stimulation in general because synaptic activity can control autophagy.^29^ Regardless of these differences, the findings from both groups provide evidence to support involvement of autophagy in LTD as a burgeoning field of research.

Our findings regarding the differential distribution of autophagosomes and LTD inducibility along the proximal-to-distal axis of dendrites highlights subcellular segregation of synaptic plasticity as a component in circuit functioning. Beyond delineating the mechanism of TAP LTD, the behavioral relevance of LTD at this synapse in adults is an intriguing question for future studies. The TAP synapse consists of projections from layer III EC neurons. EC projections to the hippocampus carry spatial information. Many hippocampus-dependent behaviors rely on spatial encoding by hippocampal place cells. Place cells can remap their place fields in response to changes in spatial context or environmental cues. It is conceivable that LTD at the TAP synapse in adulthood provides additional capacity for the EC to modify place fields beyond the tri-synaptic loop of the hippocampus.

In sum, this study demonstrates a potential link between the subcellular distribution of autophagosomes in hippocampus CA1 dendrites and the differential inducibility of LTD in the SC and TAP synapses, further contributing to the growing relationship between autophagy and LTD.

### Limitations of the study

In our preparations, we measured all autophagosomes in dendrites, those formed locally and those in transit to the soma. The 4-fold difference in autophagosomes between proximal and distal dendrites could be caused by the different capacity of autophagosome biogenesis between proximal and distal dendrites or accumulation of autophagosomes in proximal dendrites from retrograde transport. Further experiments to selectively manipulate autophagy trafficking will help to address this question. Additionally, our perturbation of caspase-3 and autophagy is at the whole-cell level from pharmacology, transgenic lines, or siRNAs. Directly altering caspase-3 at the distal dendrites would further strengthen the connection between caspase-3, autophagy inhibition, and LTD. Infusing active caspase-3 into distal dendrites would require long diffusion times and could possibly induce cell death. Future techniques to selectively activate or inhibit caspase-3 at a dendritic compartment would be required for this approach. A direct test for the causative connection between autophagy and dendritic segment-specific LTD inducibility would rely on altering autophagy function at specific synapses or recruitment of autophagosomes to distinct dendritic segments. These manipulations will also allow determination of the functional relationship between autophagosome quantity and LTD. To our knowledge, current technologies are insufficient to move autophagosomes to specific subcellular locations or manipulate the function of subpopulations of autophagosomes. Our model, where the autophagosome abundance in different dendritic segments underlies their different LTD inducibility, awaits additional support from future investigations with new technologies.

## STAR★METHODS

Detailed methods are provided in the online version of this paper and include the following:

### RESOURCE AVAILABILITY

#### Lead contact

Additional information and requests for resources and reagents should be directed to the lead contact, Zheng Li (lizheng2@nih.gov).

#### Materials availability

All materials obtained commercially have been listed in the STAR Methods key resources table. All plasmids generated by this paper, pRRLsin-RFP-LC3, pLenti-ATG5siRNA, and pLenti-SCRsiRNA can be provided upon request to the lead contact, Zheng Li.

#### Data and code availability

- Microscopy, electrophysiology, and other data will be shared by the lead contact upon request.
- This paper does not report original code.
- Any additional information required to reanalyze the data reported in this work paper is available from the lead contact upon request.

### EXPERIMENTAL MODEL AND STUDY PARTICIPANT DETAILS

Male and female C57BL/6 mice were purchased from Charles River Laboratories. The T29-1-Cre, caspase-3 knockout, and THY1-eYFP-H mice were purchased from the Jackson Laboratory. The ATG5^flox/flox^ mice were obtained from RIKEN BioResource Center.^59^ Male and female mice were used in all experiments in equal proportions. All animal procedures followed the US National Institutes of Health Guidelines Using Animals in Intramural Research and were approved by the National Institute of Mental Health Animal Care and Use Committee.

### METHOD DETAILS

#### DNA constructs and pharmacological Agents

The following constructs were purchased from Addgene: PmRFP-LC3B.^57^ The PmRFP-LC3 construct was amplified by PCR and cloned into the EcoRI/BamHI site of the pRRLsin lentiviral vector for viral production. The Atg5 siRNA (GGCTCACTTTATGTCATGT) and scrambled oligonucleotide (GACGTGAACGGATAACACT) were inserted into the BglII/HindIII site of the pSuper plasmid, in previous work^17^ and then the H1 promoter together with the siRNA sequence in the pSuper plasmid was released by restriction with XbaI and XhoI and subcloned between the same restriction sites upstream to GFP under EF1A promoter, in the pRRLsin plasmid. The following antibodies were obtained commercially: p62 (1:500 dilution for immunoblotting), LC3B (1 μg/mL for immunoblotting), GAPDH (1:100000 dilution for immunoblotting), ATG5 (1:500 dilution for immunostaining), LRRTM1 (1 μg/mL for immunoblotting) CaMKII-α (1: 500 dilution for immunostaining), NeuN (1:5000 dilution for immunostaining), anti-mouse Alexa Fluor 647 secondary antibody (1:2000 dilution for immunostaining), anti-rabbit Alexa Fluor 488 secondary antibody (1:200 for immunostaining), anti-mouse Alexa Fluor 488 secondary antibody (1:200 for immunostaining), anti-mouse HRP-conjugated secondary antibody (1:2000 dilution for western blotting), anti-rabbit HRP-conjugated secondary antibody (1:2000 dilution for western blotting), anti-sheep HRP-conjugated secondary antibody (1:2000 dilution for western blotting). The following virus was obtained from Addgene: AAV-CaMKII-ArchT-GFP^56^ and AAV-CaMKIIa-hChR2(H134R)-mCherry. The following reagents were purchased from Sigma: APV, MPEP, and Rapamycin. DCG-IV was purchased from Biotechne.

#### Western blotting

To compare the apical proximal and distal (relative to the soma) regions, hippocampal CA1 tissues were collected from P56 wild-type mice. Tissues from 4 mice were pooled for western blotting. Brains were sliced into 500 μm coronal sections with a similar protocol used to prepare acute brain slices for electrophysiology. The hippocampus was dissected out from the brain sections, and CA1 isolated under a dissecting microscope (Leica L2). The cell body layer of CA1 was cut away, then the regions containing proximal apical dendrites (25–150 μm from soma) and distal apical dendrites (200–250 μm from soma) were isolated. To compare wild-type and ATG5 knockout mice, the entire CA1 and EC regions were isolated and the tissue from each mouse was separately analyzed. Tissues were homogenized in RIPA buffer using a motorized pellet pestle and centrifuged for 20 min at 14,000 rpm at 4°C. The supernatant was removed and measured for protein concentration. The tissue lysate was diluted in SDS gel-loading buffer, separated by running a 12% SDS-PAGE gel, then transferred to a nitrocellulose membrane. The membrane was blocked with 5% milk in tris-buffered saline containing 0.1% tween (TBST) for 30 min at room temperature, followed by incubation with primary antibodies diluted in blocking buffer at 4°C overnight. The membrane was washed 5 times in TBST before being incubated with secondary antibodies for 1 h at room temperature. Membranes were washed 5 times in TBST and used for the chemiluminescence analysis with ECL (Amersham, RPN2232).

#### Lentiviral production

HEK293T cells (<3 passages) were cultured on 15-cm plates coated with 0.2% gelatin in DMEM medium supplemented with 10% fetal bovine serum until reaching 80% confluence. Fresh medium was replaced 2 h before transfection. For transfection of each 15-cm plate, 22 μg pRRLsin lentiviral vector containing RFP-LC3, 15 μg psPAX2, 5 μg pMD2.G and 2 μg pAdVantage plasmids were added to 2 mL water containing 260 μL CaCl2 (2 M). The DNA solution was added to 2 mL 2X HBSS (50 mM HEPES, 280 mM NaCl, 1.5 mM Na2HPO4, pH 7.05). After incubation at room temperature for 2 min, the mixture was added to the culture plate dropwise. The medium was replaced with 15 mL UltraCULTURE Media (UltraCULTURE, 1 mM sodium pyruvate, 0.075% sodium bicarbonate, 1x glutamine) at 16 h post-transfection. The medium was collected at 48 h after transfection and kept at 4°C. 15 mL fresh UltraCULTURE medium was added to the plate and collected at 72 h after transfection. The media collected at the two time points were combined, filtered with 0.45 μm filters, and centrifuged at 25,000 rpm for 90 min at 4°C (SW28 rotor, Beckman Coulter). The supernatant was removed and the pellet containing virus was dissolved by incubation with 100 μL 1x HBSS overnight at 4°C. For further purification of virus, the viral suspension was placed on the top of 1.5 mL 20% sucrose (in 1x HBSS) and centrifuged at 21000 rpm for 2 h at 4°C (SW55 rotor, Beckman Coulter). The pellet was incubated with 100 μL 1x HBSS overnight at 4°C, aliquoted and stored at −80°C. The titer of purified virus was determined by transducing HEK293T cells with a series of dilutions. The virus used for injection had a titer of 10^9^–10^10^ IU/mL.

#### Viral injection

4-week-old mice were anesthetized by intraperitoneal injection of Ketamine/Xylazine (Ketamine: 100 mg/kg; Xylazine: 8 mg/kg), followed by fixing the head on the stereotaxic frame. Anesthesia was maintained with isoflurane throughout surgery. Bilateral craniotomy was made above the region of interest and 1 μL either lenti or AAV virus was injected into the hippocampus CA1 (AP-1.8, ML-1.5, DV-2.0), entorhinal cortex (AP-4.3, ML-3.0, DV-3.2), or Reuniens Nucleus of the Thalamus (AP-0.6, ML-0.3, DV-4.5) with a5 μL gas-tight syringe (Hamilton, #87931, #7803-05) at a speed of 100 nL/min.

#### Tissue preparation for iSIM

4-week post lentivirus injection into CA1, mice were anesthetized with Ketamine/Xylazine (Ketamine: 100 mg/kg; Xylazine: 8 mg/kg) and perfused with ice-cold 4% paraformaldehyde (PFA) in PBS. The brain was then removed and fixed overnight in 4% PFA. The fixed brain was cryoprotected in PBS containing 15% sucrose for 1 day followed by 1 day in 30% sucrose in PBS. Cryoprotected brains were mounted coronally and cut into 30 μm sections with a Leica CM 3050 s cryostat before being mounted for imaging.

#### iSIM image acquisition

iSIM imaging was conducted on Nikon Ti body base integrated with commercial VT-iSIM (VisiTech International) module. Z-stacks of samples were acquired using a 100× oil immersion objective (NA 1.49) and sCMOS camera (ORCA-Fusion BT; Hamamatsu). Lasers, cameras, stages, triggering, and microscopy are all controlled through Nikon NIS-Elements Imaging software (version 5.30). 488-nm laser was employed to excite eYFP with a 525/50 bandpass filter inserted before the camera for collection of the emission light. 561-nm laser was employed to excite RFP with a 605/52 bandpass filter for collection of the emission light. Lateral pixel size was 65 nm in xy in Figure 1 and 46 nm in Figure S3 with 200 nm step size on acquired z-stacks.

#### iSIM image analysis

A custom Fiji script was created to analyze multichannel deconvolved iSIM images. Images were split into individual channels, and individually thresholded manually using Huang dark no-reset stack in a blinded fashion. For the RFP channel, an additional despeckle was run to ensure no improper puncta were included for colocalization. The Fiji 3D Manager suite was used to add all RFP puncta to the eYFP channel and analyze colocalization.^58^ Each RFP ROI was made up of multiple pixels, with each pixel given a binary colocalization score of 1 for colocalized with eYFP, or 0 for not colocalized with eYFP. If more than 60 percent of the RFP pixels in a specific ROI were colocalized with eYFP, then that ROI was considered to be colocalized. 3D manager was used to obtain the volume in pixels for both eYFP area and colocalized RFP puncta. Pixels were transformed to mm based on image properties.

#### Immunostaining

P56 mice anesthetized with Ketamine/Xylazine (Ketamine: 100 mg/kg; Xylazine: 8 mg/kg) were perfused with ice-cold 4% paraformaldehyde in PBS. The brain was then removed and fixed overnight in 4% PFA. The fixed brain was cryoprotected in PBS containing 15% sucrose for 1 day followed by 1 day in 30% sucrose in PBS. Cryoprotected brains were mounted horizontally and cut into 30 μm sections with a Leica CM 3050 s cryostat. Brain sections were blocked for 1 h using 5% horse serum in PBST before being incubated overnight at 4°C with a primary antibody. Sections were then washed with PBST and incubated with a secondary antibody at room temperature for 1 h. After washing, sections were mounted in mounting media for imaging.

#### Confocal image acquisition and image analysis

The Zeiss LSM 880 confocal microscope and a 20× objective (NA 0.8) was used to image brain sections. The same confocal settings were used to scan all brain sections in the same experiment. Confocal images were analyzed using ImageJ software. A z-projection was generated from the images. The 2D image was thresholded manually with the same threshold across all images in the same experiment. An ROI was drawn, around the cell body layer for CA1 and around the whole entorhinal cortex, and the raw integrated density of the ROI was measured. This value was then divided by the area of the ROI to obtain ATG5 AU/ROI Area. For quantification of mCherry positive cells, confocal images were collapsed in the z-dimension, thresholded, binarized, masked, and then counted for cell numbers with the find maxima feature in Fiji.

#### Acute hippocampal slice preparation

Young mice (16–19 days of age) or adult mice (56–70 days of age) were anesthetized with isoflurane and decapitated. The brain was removed and chilled in ice-cold sucrose cutting buffer in the case of young animals containing the following in mM (2.5 KCl, 1.25 NaH_2_PO_4_, 26 NaHCO_3_, 185 Sucrose, 25 Glucose, 20 HEPES, 5 Sodium Ascorbate, 2 Thiourea, 3 Sodium Pyruvate, 10 MgSO_4_, 0.5 CaCl_2_) or an ice-cold NMDG cutting buffer in the case of adult animals containing the following in mM (NMDG 93, KCl 2.5, NaH_2_PO_4_ 1.2, NaHCO_3_ 30, HEPES 20, Glucose 25, Sodium Ascorbate 5, Thiourea 2, Sodium Pyruvate 3, MgSO_4_ 10, CaCl_2_ 0.5 pH 7.3). 400 μm modified horizontal sections (12° angled on the ventral surface of the hemisphere from caudal to rostral^36,38^) to preserve the temporoammonic pathway were cut in ice-cold sucrose or NMDG buffer using a vibratome (VT-1000 s, Leica). Adult slices were incubated in warm (32°C) NMDG buffer for no more than 15 min and then allowed to cool down to room temperature in artificial cerebrospinal fluid (ACSF) containing in mM (124 NaCl, 2.5 KCl, 1.2 NaH_2_PO_4_, 24 NaHCO_3_, 5 HEPES, 12.5 Glucose, 2 MgCl_2_, 2 CaCl_2_ pH 7.3) for 45 min before being transferred to the recording chamber. Young slices were transferred directly to ACSF for 1 h following slicing. All solutions were continuously bubbled with 95% O_2_/5% CO_2_.

#### Electrophysiology

Slices were perfused with ACSF at 2 mL/min. One electrode was placed on the temporoammonic pathway, and another electrode was placed on the Schaffer collateral pathway. For field recordings, recording pipettes (1–2 MΩ) were filled with the bath solution and placed in the SR of CA1 for SC LTD and SLM of CA1 for all other recordings. Stimulus intensity was 60% of peak response for all experiments. LTD was induced via low-frequency stimulation (900 pulses at 1 Hz). LTP was induced via high-frequency stimulation (2 trains of 100 pulses at 100 Hz separated by 15 s). Only independent inputs passing the independent-input test and less than a 10% change in uninduced control pathway throughout the recording period were included for further analysis. The independent-input test was conducted by recording 8 sweeps 20 s apart of SC stimulation (SC1), 50 ms interpulse interval, then TAP stimulation (TAP2). The sequence was then flipped in a new recording with TAP stimulation (TAP1) taking place 50 ms before SC Stimulation (SC2). Only slices with fEPSP_SC2_/fEPSP_SC1_ and fEPSP_TAP2_/fEPSP_TAP1_ = 0.95–1.05 were included for analysis. fEPSP slope was measured from 25 to 75% of peak fEPSP amplitude in Clampfit to ensure unbiased measurements between groups.

#### Optogenetics

Animals were injected bilaterally with ArchT-GFP AAV-virus under the control of the CaMKII promoter into either the EC or NeR. Acute modified horizontal hippocampal slices were prepared at 6 weeks post-injection. An optic fiber was placed above the brain slice in the recording chamber, and a 561-nm laser (CrystaLaser) was used to stimulate ArchT. The recording electrode was placed in SLM of CA1, and an electrical stimulating electrode was placed in the TAP. In EC injected animals, to ensure ArchT was sufficiently expressed, a minimum 0.3 mV peak optically evoked post-synaptic potential was required for inclusion of the slice for further analysis. In NeR injected mice, optically evoked potentials rarely exceeded 0.1 mV, so slices with any detectable optically induced potential were included. To measure the effect of optically inhibiting the EC or NeR inputs on fEPSPs evoked by electrical stimulation of TAP, a 100-ms light pulse was applied and the TAP was electrically stimulated at 60 ms after the light onset when the optically evoked potentials were stable. Electrically induced fEPSPs in the light off and light on conditions were compared for analysis.

### QUANTIFICATION AND STATISTICAL ANALYSIS

SigmaPlot 13.0 software was used for statistical analysis. For the comparison of two independent groups or repeated groups, two-tailed Student’s t-test and two-tailed paired Student’s t-test were used respectively for data passing the normality and equal variance tests. For data failing these tests, the Mann-Whitney rank-sum test and Wilcoxon-Signed Rank test were used for two independent or repeated groups respectively. For comparison of three groups with two factors, two-way RM ANOVA was used. Data shown to be statistically significant by ANOVA were assessed post-hoc via the Holm-Sidak Method. For analysis of input output curve, SPSS was utilized to conduct a linear mixed model analysis of genotype, fiber volley, and fEPSP slope interaction. p < 0.05 was considered significant. The statistical results are described in Table S1.

## Supplementary Material

1

## Figures and Tables

**KEY RESOURCES TABLE:** 

REAGENT or RESOURCE	SOURCE	IDENTIFIER

Antibodies		

Rabbit polyclonal anti-SQSTM1/p62	Cell signaling Technology	Cat#5114; RRID: AB_10624872
Rabbit polyclonal anti-LC3B	Novus biologicals	Cat#NB100-2220; RRID: AB_10003146
Mouse monoclonal anti-GAPDH	Proteintech	Cat#60004-1-Ig; RRID: AB_2107436
Rabbit polyclonal anti-ATG5	Novus biologicals	Cat#NB110-53818; RRID: AB_828587
Sheep polyclonal anti-LRRTMl	R&D systems	Cat#AF4897; RRID:AB_10643427
Mouse monoclonal anti-CamKII-α	Cell signaling Technology	Cat#50049S; RRID: AB_2721906
Mouse monoclonal anti-NeuN	Proteintech	Cat#66836-1-Ig; RRID: AB_2882179
Goat polyclonal anti-mouse Alexa Fluor 647	ThermoFisher Scientific	Cat#A-21236; RRID: AB_2535805
Goat polyclonal anti-rabbit Alexa Fluor 488	ThermoFisher Scientific	Cat#A27034; RRID: AB_2536097
Goat polyclonal anti-mouse Alexa Fluor 488	ThermoFisher Scientific	Cat#A-11029; RRID: AB_2534088
Horse polyclonal anti-mouse-HRP linked	Cell signaling Technology	Cat#7076; RRID: AB_330924
Goat polyclonal anti-rabbit-HRP linked	Cell signaling Technology	Cat#7074; RRID: AB_2099233
Donkey polyclonal anti-sheep-HRP linked	R&D systems	Cat#HAF016 RRID:AB_562591

Bacterial and virus strains		

AAV-CaMKII-ArchT-GFP (PV2527)	Han et al.^56^	Addgene viral Prep #: 99039-AAV9; RRID: Addgene_99039
AAV-CaMKIIa-hChR2(H134R)-mCherry	A gift from Karl Deisseroth	Addgene viral Prep #: 26975-AAV9; RRID: Addgene_26975
Lenti-CaMKIIa-RFP-LC3	This paper	N/A
Lenti-ATG5siRNA-GFP	This paper	N/A
Lenti-SCRsiRNA-GFP	This paper	N/A

Chemicals, peptides, and recombinant proteins		

D-(−)-2-Amino-5-phosphonopentanoic acid (APV)	Sigma	Cat#79055-68-8
2-Methyl-6-(phenylethynyl)-pyridine hydrochloride (MPEP)	Sigma	Cat#219911-35-0
Rapamycin	Sigma	Cat#53123-88-9
(2*S*,2’*R*,3’*R*)-2-(2’,3’-Dicarboxycyclopropyl) glycine (DCG-IV)	Bio-techne	Cat#0975/10

Experimental models: Organisms/strains		

C57BL/6	Charles River	Strain code: 027
B6.Cg-Tg(Camk2a-cre)T29-1Stl/J	Jackson Laboratory	Stock number #005359; RRID: IMSR_JAX:005359
B6N.129S1-*Casp3^tm1FIv^*/J	Jackson Laboratory	Stock number #006233; RRID: IMSR_JAX:006233
B6.Cg-Tg(Thy1-YFP)HJrs/J	Jackson Laboratory	Stock number #003782; RRID: IMSR_JAX:003782
B6.129S-Atg5^tm1Myok^/MyokRbrc	RIKEN BioResource Center	Stock number #02975; RRID: IMSR_RBRC02975

Oligonucleotides		

siRNA Targeting sequence ATG5: GGCTCACTTTATGTCATGT	Shen et al.^17^	N/A
siRNA Targeting sequence SCR: GACGTGAACGGATAACACT	Shen et al.^17^	N/A

Recombinant DNA		

pmRFP-LC3	Kimura et al.^57^	Addgene Plasmid#21075; RRID: Addgene_21075
pRRLsin-RFP-LC3	This paper	N/A
pLenti-ATG5siRNA-GFP	This paper	N/A
pLenti-SCRsiRNA-GFP	This paper	N/A

Software and algorithms		

SigmaPlot 13.0	SystatSoftware	https://systatsoftware.com/
pClamp 11	Molecular Devices	moleculardevices.com
Fiji	NIH	imagej.net
Fiji 3D manager suite	Ollion et al.^58^	imagej.net/imagej-wiki-static/3D_ImageJ_Suite

## Highlights

- TAP and SC synapses have a shared mechanism of LTD reliant on autophagy inhibition
- Proximal apical dendrites have more autophagosomes than distal apical dendrites
- TAP LTD persists into adulthood, whereas SC LTD does not
