# Supplementary material for: Dendritic distribution of autophagosomes underlies pathway-selective induction of LTD
Source: Cell Rep. Author manuscript; Available in PMC 2023 Sep 27. (PMC10528062; doi:10.1016/j.celrep.2023.112898)
Supplement: 1 [file NIHMS1928252-supplement-1.pdf]

**Cell Reports, Volume *42***

## Supplemental information

# Dendritic distribution of autophagosomes underlies pathway-selective induction of LTD

**Kevin M. Keary III, Qin-Hua Gu, Jiji Chen, and Zheng Li**

Figure S1

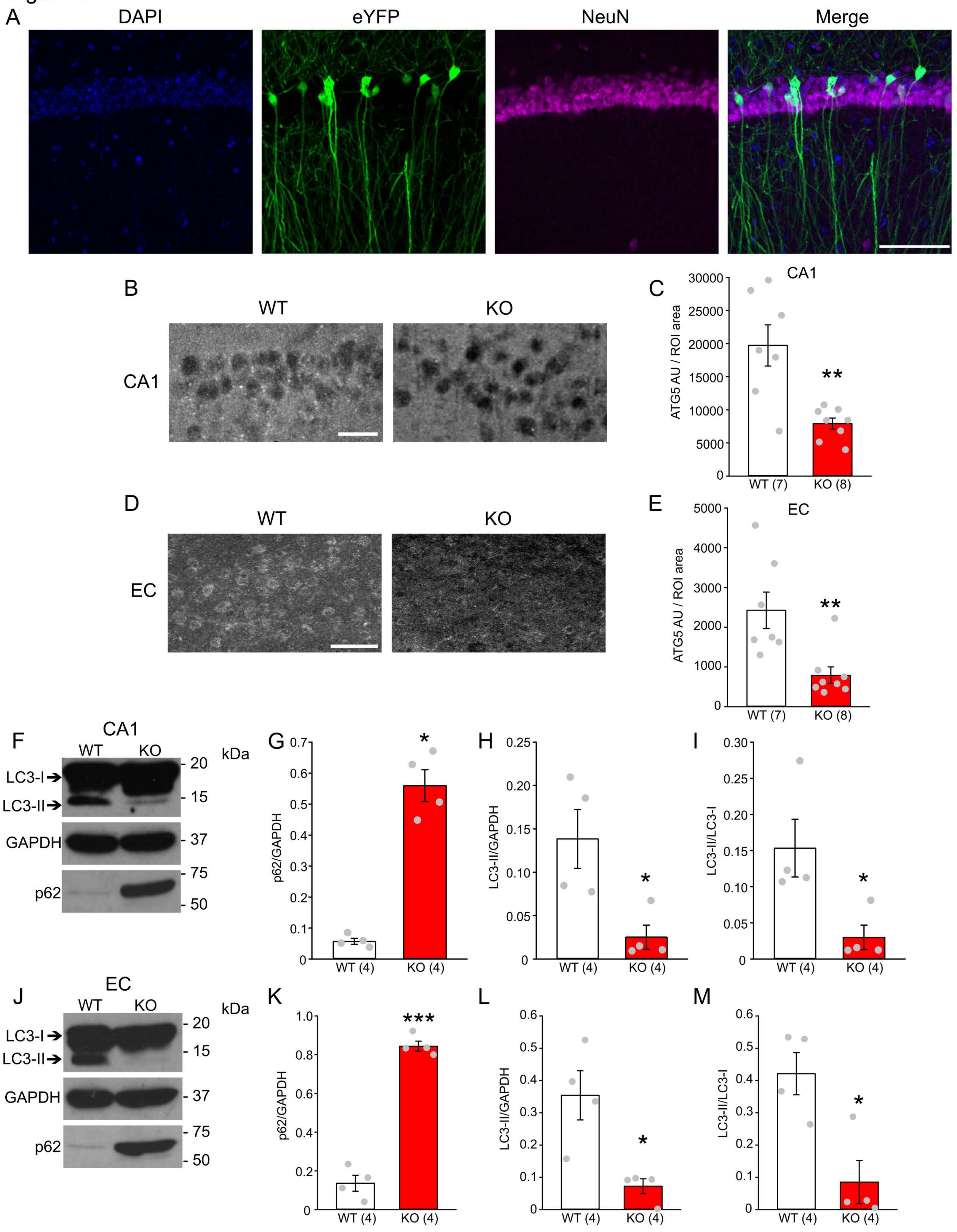

A
DAPI
eYFP
NeuN
Merge
B
WT
KO
CA1
C
CA1
ATG5 AU / ROI area
30000
25000
20000
15000
10000
5000
0
**
WT (7)
KO (8)
D
WT
KO
EC
E
EC
ATG5 AU / ROI area
5000
4000
3000
2000
1000
0
**
WT (7)
KO (8)
F
CA1
WT
KO
kDa
LC3-I
LC3-II
GAPDH
p62
20
15
37
75
50
G
p62/GAPDH
*
WT (4)
KO (4)
H
LC3-II/GAPDH
*
WT (4)
KO (4)
I
LC3-II/LC3-I
*
WT (4)
KO (4)
J
EC
WT
KO
kDa
LC3-I
LC3-II
GAPDH
p62
20
15
37
75
50
K
p62/GAPDH
***
WT (4)
KO (4)
L
LC3-II/GAPDH
*
WT (4)
KO (4)
M
LC3-II/LC3-I
*
WT (4)
KO (4)

**Figure S1: ATG5 and autophagic flux are reduced in ATG5 KO mice.**

(A) Representative confocal images of the CA1 region in THY1-eYFP mice stained with NeuN; scale bar = 100 μm. (B, D) Representative images from CA1 (B) and EC (D) of wild-type and ATG5 KO immunostaining. Scale bar is 25 μm top 50 μm bottom. (C, E) Quantification of immunostaining for wild-type and knockout slices in CA1 (C) and EC (E). n = 7 slices from 3 animals for WT and n = 8 slices from 3 animals for KO. (F) Representative blot from tissue lysates taken from the CA1 of WT and ATG5 KO mice. (G-I) Quantification of western blots for the CA1 lysates from WT and ATG5 KO mice; n = 4 biological replicates. (J) Representative blot from EC lysates of WT and ATG5 KO slices. (K-M) Quantification of western blotting; n = 4 biological replicates. Data are presented as mean ± SEM. * $p < 0.05$, ** $p < 0.01$, *** $p < 0.001$.

Figure S2

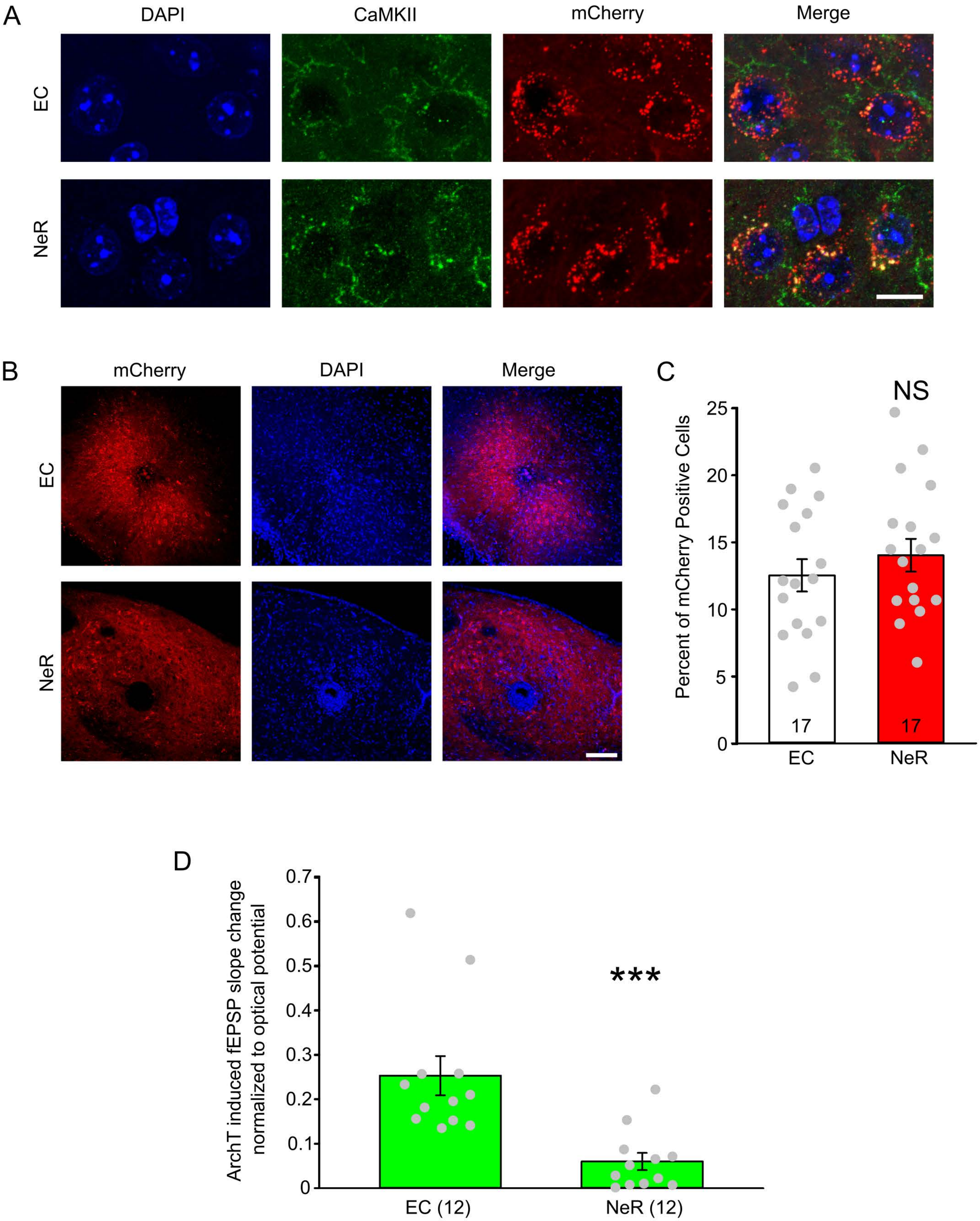

A
DAPI
CaMKII
mCherry
Merge
EC
NeR
B
mCherry
DAPI
Merge
EC
NeR
C
NS
Percent of mCherry Positive Cells
0
5
10
15
20
25
17
17
EC
NeR
D
ArchT induced fEPSP slope change normalized to optical potential
0
0.1
0.2
0.3
0.4
0.5
0.6
0.7
***
EC (12)
NeR (12)

**Figure S2: Viral infection efficiency in EC and NER injected mice.**

(A) Representative images of mCherry virus injected EC (top) and NeR (bottom) slices immunostained for CaMKII. Scale bar = 10 µm. (B) Representative images from animals injected with mCherry virus at the EC (top) and NeR (bottom); note the lesion site in the middle; scale bar = 100 µm. (C) Quantification of mCherry positive cells in EC and NeR injected animals; n = 17 images from 3 animals. (D) Normalization of ArchT-induced fEPSP change to optical potentials; n = 12 slices from 7 animals for EC and n = 12 slices from 6 animals for NeR. Each data point represents the average change in fEPSP slope normalized to optical potentials from each slice. Data are presented as mean ± SEM. *** $p < 0.001$.

Figure S3

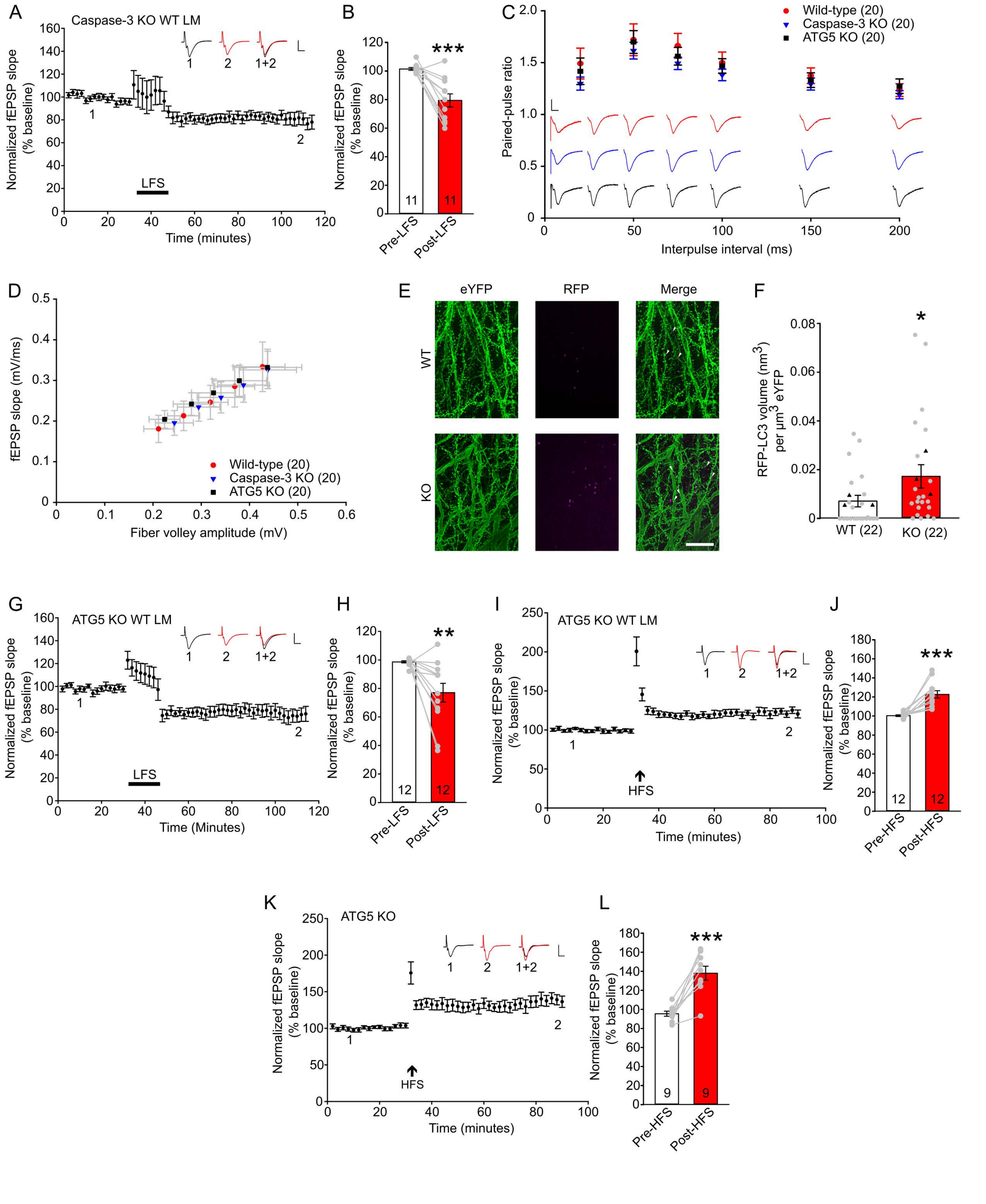

A
Caspase-3 KO WT LM
Normalized fEPSP slope (% baseline)
LFS
Time (minutes)
1
2
1+2
B
***
Pre-LFS
Post-LFS
11
C
Paired-pulse ratio
Interpulse interval (ms)
Wild-type (20)
Caspase-3 KO (20)
ATG5 KO (20)
D
fEPSP slope (mV/ms)
Fiber volley amplitude (mV)
Wild-type (20)
Caspase-3 KO (20)
ATG5 KO (20)
E
eYFP
RFP
Merge
WT
KO
F
*
RFP-LC3 volume (nm$^3$) per µm$^3$ eYFP
WT (22)
KO (22)
G
ATG5 KO WT LM
Time (Minutes)
H
**
12
I
ATG5 KO WT LM
HFS
Time (minutes)
J
***
Pre-HFS
Post-HFS
K
ATG5 KO
L
9

**Figure S3: LTD, LTP, PPR, and input-output relationship in ATG5 knockout and wild-type littermates of caspase-3 knockout and ATG5 knockout mice.**

(A, B) Normalized fEPSPs evoked in slices from adult wild-type littermates (LM) of caspase-3 knockout mice and quantification of fEPSPs before and after LFS; n = 11 slices from 11 animals. (C) Paired-pulse ratio measured by stimulating TAP in wild-type, ATG5 KO, and caspase-3 KO slices; data points are averaged PPR from 20 slices from 6 animals. (D) Input-output curve measured by stimulating TAP in wild-type, ATG5 KO, and caspase-3 KO mice; data points are averaged fiber volley and fEPSP slope from 20 slices from 6 animals. (E) Representative iSIM images of CA1 apical distal dendrites from THY1-eYFP WT (top) and THY1-eYFP-caspase-3 knockout mice (bottom) injected with the RFP-LC3 virus; white arrow heads indicate colocalized RFP-LC3 puncta in eYFP-labeled dendrites; scale bar: 10 µm. (F) Quantification of RFP-LC3 puncta volume in eYFP-labeled neurons; n = 22 images from 3 animals black triangles denote animal means. (G, H) fEPSPs evoked in adult ATG5 knockout wild-type littermate slices and quantification of fEPSPs before and after LFS; n = 12 slices from 8 animals. (I, J) Normalized fEPSPs evoked in slices from the wild-type littermates of adult ATG5 knockout mice and quantification of fEPSPs before and after HFS; n = 12 slices from 7 animals. (K, L) fEPSPs evoked in adult ATG5 knockout slices and quantification of fEPSPs before and after HFS; n = 9 slices from 4 animals. Each data point in A, G, I, and K represents averaged response from all slices of 4 consecutive traces evoked every 30 seconds; scale bar is 0.5 mV vertical and 20 ms horizontal. Each data point in B, H, J, and L represents the average of first 10 minutes of pre-induction and last 10 minutes of post-induction from each slice. Data are presented as mean ± SEM. * $p < 0.05$, ** $p < 0.01$, *** $p < 0.001$.

Figure S4

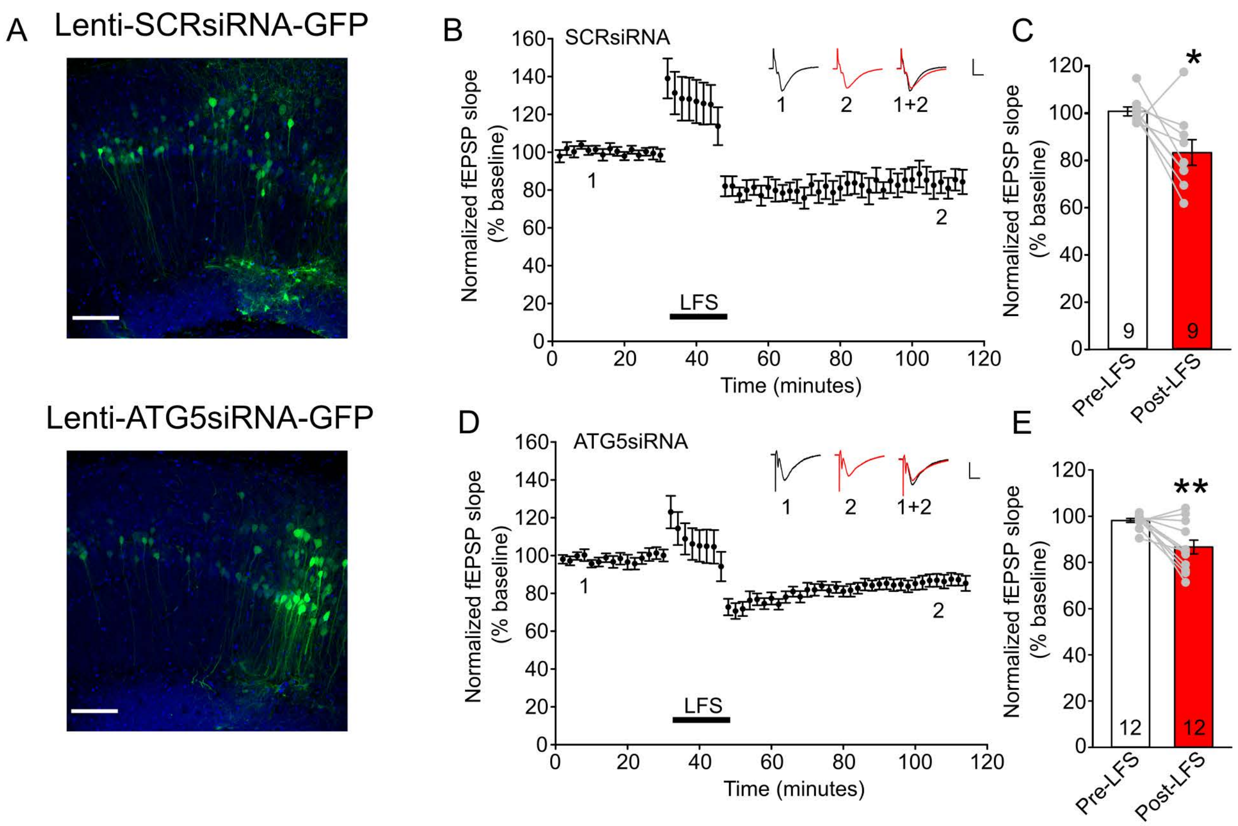
A
Lenti-SCRsiRNA-GFP
Lenti-ATG5siRNA-GFP
B
SCRsiRNA
Normalized fEPSP slope (% baseline)
Time (minutes)
LFS
1
2
1+2
C
Normalized fEPSP slope (% baseline)
Pre-LFS
Post-LFS
9
9
*
D
ATG5siRNA
Normalized fEPSP slope (% baseline)
Time (minutes)
LFS
1
2
1+2
E
Normalized fEPSP slope (% baseline)
Pre-LFS
Post-LFS
12
12
**

**Figure S4: ATG5 knockdown in postsynaptic neurons has no effect on LTD.**

(A) Representative images of slices from adult animals injected with Lenti-SCRsiRNA-GFP virus (top) and Lenti-ATG5siRNA-GFP virus (bottom) in CA1. Scale bar = 100 µm. (B, C) Normalized fEPSPs evoked in SCRsiRNA virus injected slices and quantification before and after LFS; $n = 9$ slices from 6 animals. (D, E) fEPSPs evoked in adult ATG5siRNA injected slices and quantification before and after LFS; $n = 12$ slices from 5 animals. Each data point in B and D represents averaged response from all slices of 4 consecutive traces evoked every 30 seconds. Scale bar is 0.5 mV vertical and 20 ms horizontal in B and D. Each data point in C and E represents the average of first 10 minutes of pre-LFS and last 10 minutes of post-LFS from each slice. Data are presented as mean ± SEM. * $p < 0.05$, ** $p < 0.01$.

Table S1: Summary Table of Statistical Analysis

| Figure | Model | Results | |
|---|---|---|---|
| | | Model Term | p-value |
| 1C | Mann-Whitney Rank Sum | Wild Type vs. Knockout | <0.001 |
| 1F | Mann-Whitney Rank Sum | Proximal vs. Distal | <0.001 |
| 1H | Two-Tailed T-Test | Proximal v Distal LRRTM1/GAPDH | 0.000795 |
| 1J | Mann-Whitney Rank Sum | Proximal vs. Distal p62/GAPDH | 1 |
| 1K | Mann-Whitney Rank Sum | Proximal vs. Distal LC3-II/GAPDH | 0.029 |
| 1L | Two Tailed T-Test | Proximal vs. Distal LC3-II/LC3-I | 0.000242 |
| 2H | Paired Two-Tailed T-Test | EC Light Off vs. EC Light On | 0.000000129 |
| 2I | Wilcoxon Signed Rank Test | NeR Light Off vs. NeR Light On | 0.064 |
| 2J | Paired Two-Tailed T-Test | TAP Pre v Post DCG-IV | 0.00000126 |
| 2K | Wilcoxon Signed Rank Test | SC Pre v Post DCG-IV | 0.375 |
| 3C | Wilcoxon Signed Rank Test | Young SC Pre LFS vs. Post LFS | <0.001 |
| 3E | Paired Two-Tailed T-Test | Adult SC Pre LFS vs. Post LFS | 0.386 |
| 3G | Paired Two-Tailed T-Test | Young TAP Pre LFS vs. Post LFS | 0.00349 |
| 3I | Paired Two-Tailed T-Test | Adult TAP Pre LFS vs. Post LFS | 0.0335 |
| 3K | Paired Two-Tailed T-Test | Adult APV Pre LFS vs. Post LFS | 0.166 |
| 3M | Paired Two-Tailed T-Test | Adult MPEP Pre LFS vs. Post LFS | 0.00153 |
| 3N | Two-Way RM ANOVA | LTD Phase | 0.997 |
| | | Interpulse Interval | <0.001 |
| | | Interaction | 0.596 |
| 4B | Paired Two-Tailed T-Test | Adult Caspase-3 KO Pre LFS vs. Post LFS | 0.879 |
| 4D | Paired Two-Tailed T-Test | Adult Rapamycin Pre LFS vs. Post LFS | 0.589 |
| 4F | Paired Two-Tailed T-Test | Adult Rapamycin ATG5 KO Pre LFS vs. Post LFS | 0.0000883 |
| 4H | Wilcoxon Signed Rank Test | Adult ATG5 KO Pre LFS vs. Post LFS | <0.001 |
| 4J | Paired Two-Tailed T-Test | SCRsiRNA+Rap Pre-LFS v Post-LFS | 0.397 |
| 4L | Paired Two-Tailed T-Test | ATG5siRNA+Rap Pre-LFS v Post-LFS | 0.00844 |
| S1C | Mann-Whitney Rank Sum | WT vs. ATG5 cKO CA1 ATG5 AU/ROI Area | 0.009 |
| S1E | Mann-Whitney Rank Sum | WT vs. ATG5 cKO EC ATG5 AU/ROI Area | 0.004 |
| S1G | Mann-Whitney Rank Sum | CA1 WT vs. KO p62/GAPDH | 0.029 |
| S1H | Mann-Whitney Rank Sum | CA1 WT vs. KO LC3-II/GAPDH | 0.029 |
| S1I | Mann-Whitney Rank Sum | CA1 WT vs. KO LC3-II/LC3-I | 0.029 |
| S1K | Two-Tailed T-Test | EC WT vs. KO p62/GAPDH | 0.00000657 |
| S1L | Two-Tailed T-Test | EC WT vs. KO LC3-II/GAPDH | 0.0122 |
| S1M | Two-Tailed T-Test | EC WT vs. KO LC3-II/LC3-I | 0.0116 |
| S2C | Two-Tailed T-Test | EC v NeR % mCherry Positive Cells | 0.279 |
| S2D | Mann-Whitney Rank Sum | EC v NeR ArchT fEPSP slope change normalized to ArchT Pulse | <0.001 |
| S3B | Paired Two-Tailed T-Test | Caspase-3 WT Pre-LFS v Post-LFS | 0.000701 |
| S3C | Two-Way RM ANOVA | Transgene | 0.624 |
| | | Interpulse Interval | <0.001 |
| | | Interaction | 0.475 |
| S3D | Linear Mixed Model Analysis | fEPSP*Fiber Volley vs. Genotype | 0.326 |
| S3F | Mann-Whitney Rank Sum | WT Distal v Caspase-3 Distal | 0.021 |
| S3H | Paired Two-Tailed T-Test | ATG5 WT LM LTD Pre-LFS v Post-LFS | 0.00784 |
| S3J | Paired Two-Tailed T-Test | ATG5 WT LM LTP Pre-HFS v Post-HFS | 0.00023 |
| S3L | Paired Two-Tailed T-Test | ATG5KO LTP Pre-HFS v Post-HFS | 0.000235 |
| S4C | Paired Two-Tailed T-Test | SCRsiRNA Pre-LFS v Post-LFS | 0.0211 |
| S4E | Paired Two-Tailed T-Test | ATG5siRNA Pre-LFS v Post-LFS | 0.00355 |
